# A holistic approach to promoting early child development: a cluster randomised trial of a group-based, multicomponent intervention in rural Bangladesh

**DOI:** 10.1136/bmjgh-2020-004307

**Published:** 2021-03-16

**Authors:** Helen O Pitchik, Fahmida Tofail, Mahbubur Rahman, Fahmida Akter, Jesmin Sultana, Abul Kasham Shoab, Tarique Md. Nurul Huda, Tania Jahir, Md Ruhul Amin, Md Khobair Hossain, Jyoti Bhushan Das, Esther O Chung, Kendra A Byrd, Farzana Yeasmin, Laura H Kwong, Jenna E Forsyth, Malay K Mridha, Peter J Winch, Stephen P Luby, Lia CH Fernald

**Affiliations:** 1 Division of Epidemiology, School of Public Health, University of California, Berkeley, Berkeley, California, USA; 2 Nutrition and Clinical Services Division, International Centre for Diarrhoeal Disease Research Bangladesh, Dhaka, Dhaka District, Bangladesh; 3 Infectious Diseases Division, International Centre for Diarrhoeal Disease Research Bangladesh, Dhaka, Dhaka District, Bangladesh; 4 Department of Epidemiology, Gillings School of Public Health, University of North Carolina at Chapel Hill, Chapel Hill, North Carolina, USA; 5 WorldFish, Bayan Lepas, Penang, Malaysia; 6 Woods Institute for the Environment, Stanford University, Stanford, California, USA; 7 Center for Non-communicable Diseases and Nutrition, BRAC James P Grant School of Public Health, BRAC University, Dhaka, Dhaka District, Bangladesh; 8 International Health, Johns Hopkins Bloomberg School of Public Health, Baltimore, Maryland, USA; 9 Division of Infectious Diseases and Geographic Medicine, Stanford University, Stanford, California, USA; 10 Division of Community Health Sciences, School of Public Health, University of California, Berkeley, Berkeley, California, USA

**Keywords:** cluster randomized trial, maternal health, child health, prevention strategies

## Abstract

**Introduction:**

In low- and middle-income countries, children experience multiple risks for delayed development. We evaluated a multicomponent, group-based early child development intervention including behavioural recommendations on responsive stimulation, nutrition, water, sanitation, hygiene, mental health and lead exposure prevention.

**Methods:**

We conducted a 9-month, parallel, multiarm, cluster-randomised controlled trial in 31 rural villages in Kishoreganj District, Bangladesh. Villages were randomly allocated to: group sessions (‘group’); alternating groups and home visits (‘combined’); or a passive control arm. Sessions were delivered fortnightly by trained community members. The primary outcome was child stimulation (Family Care Indicators); the secondary outcome was child development (Ages and Stages Questionnaire Inventory, ASQi). Other outcomes included dietary diversity, latrine status, use of a child potty, handwashing infrastructure, caregiver mental health and knowledge of lead. Analyses were intention to treat. Data collectors were independent from implementers.

**Results:**

In July–August 2017, 621 pregnant women and primary caregivers of children<15 months were enrolled (group n=160, combined n=160, control n=301). At endline, immediately following intervention completion (July–August 2018), 574 participants were assessed (group n=144, combined n=149, control n=281). Primary caregivers in both intervention arms participated in more play activities than control caregivers (age-adjusted means: group 4.22, 95% CI 3.97 to 4.47; combined 4.77, 4.60 to 4.96; control 3.24, 3.05 to 3.39), and provided a larger variety of play materials (age-adjusted means: group 3.63, 3.31 to 3.96; combined 3.81, 3.62 to 3.99; control 2.48, 2.34 to 2.59). Compared with the control arm, children in the group arm had higher total ASQi scores (adjusted mean difference in standardised scores: 0.39, 0.15 to 0.64), while in the combined arm scores were not significantly different from the control (0.25, –0.07 to 0.54).

**Conclusion:**

Our findings suggest that group-based, multicomponent interventions can be effective at improving child development outcomes in rural Bangladesh, and that they have the potential to be delivered at scale.

**Trial registration number:**

The trial is registered in ISRCTN (ISRCTN16001234).

**Key questions**

**What is already known?**

- Caregiving interventions that include responsive stimulation improve early child development outcomes
- Water, sanitation and hygiene (WASH), nutrition, caregiver mental health and lead exposure prevention are important contributors to early child development outcomes.

**What are the new findings?**

- A child stimulation intervention that includes integrated contents on WASH, nutrition, caregiver mental health and lead exposure prevention, is feasible to deliver to mixed groups of pregnant women and primary caregivers of children under 24 months of age.
- This intervention improves stimulating caregiving behaviours and child development and shows the potential for impact across multiple other risk factors for poor child development.

**What do the new findings imply?**

- Interventions to improve early child development that integrate components on multiple risk factors for child health and development should be considered as an alternative to siloed interventions.
- Research on the impacts of multicomponent interventions on outcomes in middle and late childhood is needed to determine if these initial effects are sustained.

## Introduction

Early motor, cognitive and socioemotional development affect later life outcomes, including educational attainment and economic earnings.^1^ In low- and middle-income countries (LMICs), children experience a disproportionally high burden of risk factors for delayed development when compared with children in high-income countries. An estimated one-third of 3-year-old and 4-year-old children in LMICs—80.8 million children in total—did not meet basic developmental milestones in 2010.^2^ Factors that promote development during early life include responsive caregiving, maternal and child nutrition, caregiver’s mental health, exposure to opportunities for early learning and avoidance of infection.^3^

Across many different countries, cultures and contexts, caregiver-support programmes have improved short-term early child development (ECD) outcomes by encouraging responsive caregiving and stimulation through the promotion of age-appropriate caregiver-child interactions.^4^ Interventions addressing other risk factors for poor child development including maternal mental health, nutrition and water, sanitation and hygiene (WASH) have also been shown to improve parental investments for children,^5^ or ECD outcomes,^6 7^ though effects are smaller than for interventions that include responsive stimulation. Additionally, lead exposure has been associated with impaired cognitive development and can occur through exposure to contaminated turmeric and lead-soldered food storage cans.^8–10^ Globally up to 800 million children, mostly in LMICs, have elevated lead exposure, but interventions have not assessed the impact of lead exposure reduction on ECD outcomes.^11^

Integrated interventions targeting multiple risk factors have been recommended in the WHO guideline for improving ECD outcomes.^12^ Integration of contents on multiple components may allow for efficiencies in intervention delivery through economies of scope, and may result in more holistic changes in the early environment, resulting in improved outcomes and cost savings. For example, an integrated stimulation, nutrition and health intervention in rural China showed positive effects on multiple outcomes beyond child development, including caregiver-reported child health, nutrition and diarrhoea prevalence.^13^ However, the evaluation of an intervention in rural India demonstrated that it is possible that integrating many intervention components may take caregivers’ focus away from stimulating caregiving practices and disperse behaviour changes across multiple domains.^14^

Sustainability and scalability of ECD interventions are critical to their ultimate success, and this has driven the push to explore group-based delivery mechanisms. Many responsive stimulation interventions were originally developed to be delivered in home visits, which allow for more personalised coaching and problem-solving when compared with group sessions; however, delivery at-scale may be easier to attain with groups.^15^ Group-based intervention delivery may also improve maternal mental health by facilitating the development of formalised social support networks in the community, and may contribute to sustained changes in community norms.^16^ Thus, groups may promote longer term intervention effects, an important consideration given the recent demonstrations of a fade-out of initial promising effects of scaled-up home-visiting programmes.^17^

In this study, we aimed to evaluate the effects of a multicomponent group-based responsive stimulation, nutrition, WASH, maternal mental health and lead exposure prevention intervention in rural Bangladesh on stimulating caregiving practices, child development and multiple other risk factors for poor child development. We tested two delivery mechanisms, one that consisted of only group sessions and one that combined group sessions and home visits. We hypothesised that the integrated multicomponent intervention would improve caregiving practices, child development and caregiver mental health through both delivery mechanisms.

## Methods

### Study design and participants

The Research on Integrated Nutrition, ECD and WASH (RINEW) intervention was a three-arm cluster-randomised controlled trial conducted in the Katiadi and Kuliarchar subdistricts of Kishoreganj District, Bangladesh. Trial arms were (1) community group sessions (group arm), (2) alternating community group sessions and home visits (combined arm) and (3) passive control. As group sessions were community based, villages were used as the unit of randomisation to avoid spillover of intervention contents across arms.

All villages in the Katiadi and Kuliarchar subdistricts with populations between 200 and 800 households were considered for inclusion except for those in Masua union, where formative work was conducted. Villages were excluded if their basic demographic factors (ie, literacy, electricity status) were more than 1.5 standard deviations (SDs) higher or lower than district averages. This was done to decrease the probability of chance imbalances in the intervention arms at baseline, which would decrease precision of effect estimates. Remaining villages were included if they were at least 2 km apart. Exceptions to these criteria are described in the supplementary material (online supplemental table S1).

10.1136/bmjgh-2020-004307.supp1 Supplementary data

Eligible participants were women living in the selected villages who were in their second or third trimester of pregnancy or primary caregivers of a child under 15 months of age. All participants were eligible for all 18 intervention sessions. The pregnant woman’s in-utero child, or the youngest child of the primary caregiver (for participants who were not pregnant) was considered to be the child enrolled in the RINEW study. All participants gave verbal and written informed consent before being enrolled in the study.

### Randomisation and masking

Each village was a cluster, and randomisation was stratified by subdistrict. Clusters within each subdistrict were randomly allocated into one of two active intervention arms, or an oversized control arm, by an investigator at the University of California, Berkeley (HOP). The allocation ratio was 5:5:8 in Katiadi, and 3:3:7 in Kuliarchar for the group, combined and control arms, respectively. We used an oversized control arm to improve precision when comparing each intervention arm to the control arm. Participants were randomly selected from eligible participants in each cluster. Participants were informed of their intervention assignment following the baseline survey. Study participants and community health workers (CHWs) were not masked to intervention arm as the control arm participants were not invited to sessions with CHWs and only the combined arm included household visits. To mask data collectors to group status, they were independent from CHWs and were not made aware of the study design or intervention components. Though items from the intervention sessions (toys, books) may have been visible to data collectors, they were not made aware that these contents were part of the intervention.

### Intervention

The RINEW intervention took place between September 2017 and May 2018. All participants in villages randomised to either the group or combined intervention arms were invited to attend 18 intervention sessions delivered by CHWs every 2 weeks for 9 months. The integrated multicomponent intervention curriculum was developed through a year-long piloting process. Each of the individual intervention components was developed and refined by adapting existing curricula based on field testing and feedback from community members and CHWs.^18^ Group sessions took 45-60 min and home visits took 20-25 min. Those in the group arm received 18 group sessions delivered every 2 weeks in a location close to their homestead with 3-6 pregnant women and caregiver-child dyads. Those in the combined arm received nine group sessions alternating with nine individual home visit sessions, with an intervention session every 2 weeks. Groups were assembled based on geographic proximity. The material covered was equivalent across the delivery mechanisms. In home visit sessions, facilitators discussed the age-specific recommendations presented in the group sessions that were applicable to the household. CHWs did not visit the control communities.

Each intervention session included age-specific material on responsive stimulation. For caregivers with children this portion included a brief interactive discussion about the importance of play, review of activities from previous sessions, the introduction of new developmentally appropriate games with low-cost toys made from recycled materials, a local song and activities with a simple picture book. The main aim of the stimulation component in each session was to encourage caregivers to participate in responsive caregiving and create learning opportunities through positive interaction, and to teach pregnant women how to engage in responsive stimulation with their newborn children. This intervention component was adapted from the Jamaican Reach Up Programme,^19^ with materials added for pregnant women and caregivers with children under 6 months of age. Each session also included material on one or more of the integrated components which included nutrition, WASH, lead exposure prevention and caregiver mental health (table 1, online supplemental table S2). A tablet application was used to guide CHWs through the age-relevant curriculum depending on who was recorded present in each session, using the CommCare software platform. Pregnant participants were encouraged to watch and learn from the activities conducted with caregiver-child dyads.

**Table 1:** Intervention components

Component	Description of the intervention components delivered in the group and combined intervention arms
Nutrition	Contents: This component was adapted from the WASH-Benefits intervention.^6^ The nutrition component included specific recommendations for each trimester of pregnancy, recommendations for lactation and recommendations for the complementary feeding period. Supplements: Nutritional supplements were distributed to participants depending on age and child nutritional status as indicated by mid-upper arm circumference (MUAC). Pregnant women and caregivers of children under 6 months of age were given multiple micronutrients Children with a MUAC 11.5–12.49 cm were given small-quantity lipid-based supplements (SonaMoni) Children over 6 months with a MUAC 12.5 cm and greater were given multiple micronutrient powder (Pushtikona) Children over 6 months with a MUAC under 11.5 cm were referred to a health facility (control arm children were also referred)
WASH	This component was adapted from the WASH-Benefits intervention and included activities to coach participants to identify changes they could make in their own environments.^6^ Soapy water bottles were provided to all households.
Lead	This component included teaching participants about the harms of lead and encouraging changes in their household to reduce lead exposure from previously identified lead sources: turmeric and lead-soldered cans.^10 36^
Mental health	This component was adapted from the thinking healthy programme.^37^ Through field piloting the strategies were simplified, integrated with other intervention material and incorporated behavioural activation.^18^
Targeted households	Participants with a MUAC under 12.5 were considered at risk: 17 at-risk participants who did not have access to their own hygienic latrine at baseline received WASH infrastructure (a child potty, a handwashing station and a dual pit latrine). Pregnant women received foot measurement sticks: participants who gave birth to a child who had a foot length <7 cm received a visit from the CHW who confirmed the foot length and provided (1) a session to teach the mother to provide Kangaroo Mother Care (KMC) to the baby; and (2) gave the mother a KMC kit that included three KMC pouches, one hat and one pair of socks.
Toys and books	All participants in sessions received low-cost picture books and toys made out of recycled materials for children over 6 months of age

WASH, water, sanitation and hygiene.

For sessions 9 and onwards, other caregivers were invited to attend sessions, with a focus on assisting with childcare during the parts of the session not focused on caregiver-child interaction. In addition, concurrently with the 15th and 16th intervention sessions fathers were invited to attend two separate group sessions with 10–12 peers. These sessions primarily focused on components that required support from household decision makers, including upgrading WASH infrastructure, purchasing lead-free food storage containers and unpolished turmeric and improving the diversity of food purchased for the household.

CHWs were 18-38 year-old women (mean 28 years) from the selected villages who had completed secondary school education. Many of the CHWs (75%) had previously worked in education or health. CHWs received 8 days of basic training, 4 days of refresher and tablet training immediately prior to the start of the intervention and 9 additional 2–3 day trainings during the 9-month intervention. Trainings included didactic sessions, in-class practice and field practice where CHWs were given feedback and practiced observing and giving feedback to others. At least one group session (or three individual home visits) per CHW was supervised during each 2-week period. Supervisors filled out session monitoring sheets and provided feedback to CHWs.

### Assessments

After enrolment, baseline data were collected on demographic information for all participants and child-related measures for children over 6 months of age. A team of university-educated enumerators who were not involved in intervention delivery conducted endline data collection during two home visits immediately following intervention completion. The first visit included assessments of the home environment, child development and maternal mental health, and enumerators received 12 days of training; the second visit included assessements of WASH, nutrition and lead and enumerators received 6 days of training. Training for both modules included interactive discussion, role play and field testing in non-intervention sites followed by interobserver reliability testing, feedback and refresher trainings. Interviews were conducted in Bengali, and data was collected using a tablet computer with CommCare software.

### Outcomes

The pre-specified primary quantitative outcome of this trial was the Family Care Indicators (FCI), a caregiver report questionnaire with an observation component used to assess stimulation in the home.^20^ This outcome contains two primary subscales, stimulating caregiving practices and the variety of play materials available in the home. The stimulating caregiving practices subscale has questions about the variety of stimulating caregiving activities that any adult has engaged with the child in the previous 3 days (six items). We analysed data on stimulation provided by the primary caregiver who was invited to attend the intervention sessions. The variety of play materials subscale includes observations of the variety of play materials in the home that the caregiver reported the child played with in the previous 30 days (six items). During the FCI interview caregiver responsiveness and the child’s environment were observed and recorded. This observation scale includes items from the Infant Toddler Home Observation for Measurement of the Environment about caregiver responsiveness and interactions with the child and two items on the safety of the home environment (online supplemental table S3).^21^ The prespecified secondary outcome was child development as assessed by the Ages and Stages Questionnaire Inventory (ASQi). The ASQi is primarily a caregiver report measure used to assess attainment of milestones in the communication, gross motor, fine motor, problem-solving and personal social domains of development for children between 1 and 54 months. The ASQi includes direct assessment items for a subset (50) of the questions across five domains (online supplemental table S4). The ASQi was piloted by our study team on 60 children not included in this study sample, to ensure appropriate ranking of questions. In addition, an inventory developed following the principles of the MacArthur-Bates Communicative Development Inventories (CDI) was used to capture language development in both the expressive and receptive domains. Raw ASQi and CDI scores for each domain were internally age-standardised to the control arm using age-conditional means and SDs. Children with standardised scores over 4 were excluded. Total ASQi scores were created by summing raw scores across the five domains before standardising.

Other outcomes included maternal dietary diversity assessed using the Minimum Dietary Diversity for Women score, an indicator of adequate dietary diversity when at least 5 of 10 food groups are consumed in the previous 24 hours.^22^ Dietary diversity in young children is a similar indicator, and the cut-off for achieving the minimum is the consumption of at least 5 of 8 food groups, including breastmilk.^23^ Maternal depressive symptoms were measured with the 20-question Center for Epidemiologic Studies Depression scale (CES-D). Maternal depressive symptoms scores were analysed with the continuous 60-point CESD-D score. Maternal knowledge about lead was assessed by asking if respondents had ever heard of lead, and household WASH status was assessed through the observed presence of a handwashing station with water and soap or a soapy water bottle and of a clean, functional, hygienic latrine in the household.

Ongoing inter-rater reliability was conducted during the endline assessment for 4.7% of the sample (n=27). Inter-rater reliabilities for the ASQi domains and the home observation subscale were high (the intraclass correlation for ASQi domains was ≥0.99 for all domains except for personal social, where it was 0.93; for the home observation subscale the ICC was 0.92)

As a supplementary analysis, we collected data on a direct-assessment measure of child development, the Bayley Scales of Infant and Toddler Development, Third Edition (Bayley-III), for a stratified random subset of 16 villages from those that had children of both sexes in each age group (8 control, 4 group, 4 combined); 254 children (n=134 control; n=120 intervention) were randomly selected after stratifying by age group (6–12, 13–18 and 19–24 months) and sex.

### Statistical analysis

Sample size calculations were conducted for the total FCI score (range 0–13), based on a difference of 2.0 in mean total score between each intervention arm and the control arm and an SD of 3.3.^24^ The calculations assumed an intracluster correlation of 0.20, power of 0.80 and type 1 error of 0.05. With 20 participants per cluster, the sample size calculations indicated that 15 control arm villages and 8 villages in each of the intervention groups were required. The study was not powered to detect differences between the two intervention delivery methods.

All analyses were conducted according to the randomised intervention arm at enrolment (intention to treat), without considering session attendance. The primary analysis consisted of age-adjusted mean differences between the control arm and each of the group and combined intervention arms and at endline for the primary outcomes (FCI play activities and play materials subscales). Secondary analyses include mean differences (for continuous outcomes) or prevalence differences (for binary outcomes) for child development and other outcomes. Potential covariates for inclusion in adjusted models were selected based on the child development literature, and included parental education, child age and sex, household income, household wall material, household assets and the outcome of interest measured at baseline. Interviewer was also included as a potential covariate in the adjustment set for child development and observed home environment outcomes. For each outcome, covariates were prescreened using a likelihood ratio test, and all covariates with p<0.20 were included in adjusted analyses. Adjusted analyses were done with parametric g-computation using linear regression for continuous outcomes and logistic regression for binary outcomes to generate mean differences and prevalence differences for these outcomes, respectively.^25^ CIs were generated with bootstrapped samples clustered by village (1000 samples). For each outcome except for the Bayley-III, two comparisons to the control arm were made, one for each intervention arm. For the Bayley-III assessment, only one comparison to the control arm was made, with children in any intervention arm combined due to small sample sizes. No adjustments were made for multiple comparisons.^26^ Analyses were performed in Stata V.14 and R (V.4.0.1, Vienna, Austria), with the riskCommunicator package.^27^

### Role of the funding source

This research was funded by the Bill and Melinda Gates Foundation. The funders approved the study design, but did not play a role in data collection, analysis, or interpretation of the data, in the writing of the report, or in the decision to submit the article for publication. The corresponding author had full access to the data for this study and had final responsibility for the decision to submit for publication.

### Patient and public involvement

Feedback from community members was used to adapt and refine intervention components prior to intervention delivery.

## Results

**Figure 1:**
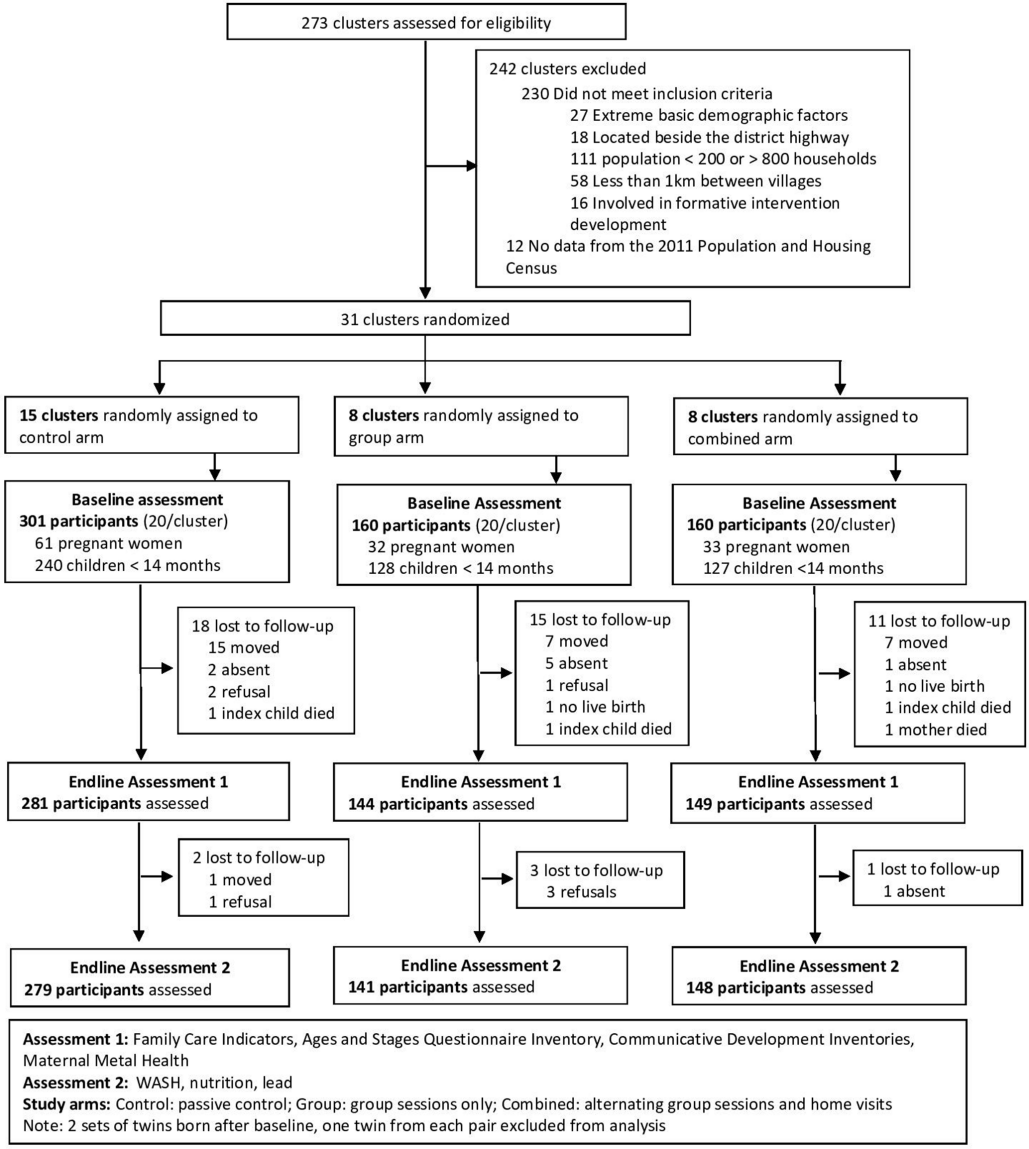
Trial profile.

Between July and August 2017, fieldworkers enrolled 621 pregnant women and primary caregivers of children under 15 months of age located in 31 villages in the RINEW trial. At intervention endline, 47 (7.6%) participants were lost to follow-up, and 6 participants had only 1 day of data collection complete, resulting in full data collection on 574 (91.4%) participants (figure 1); the majority of those lost had migrated. Loss to follow-up was not statistically significantly different across study arms (control 6.6%; group 10.0%; combined 6.9%), or demographic variables collected from participants at baseline. Intervention arms were similar when compared with the control arm across many baseline values (table 2). At intervention endline, the mean age of the children assessed was 16.5 months (range 3.9–26.4, SD 5.4), and the primary caregiver was the target child’s mother for 570 (99%) participants (4 interviews were done with other female primary caregivers of the child).

**Table 2:** Characteristics of the sample at baseline

	Study arm, n (%) or mean±SD		
	Control (n=301)	Group (n=160)	Combined (n=160)
Caregiver characteristics			
Age	25±5.6	25±6.4*	25±6.2
Completed primary education	173 (57%)	86 (54%)	101 (63%)
Pregnant woman enrolled	61 (20%)	32 (20%)	33 (21%)
CES-D score (0–60)	12.4±8.6	12.8±8.7*	13.4±9.7
Knowledge of lead	67 (22%)	47 (29%)	49 (31%)
Child characteristics (n=496)			
Age (in months)	7.0±3.9	6.7±3.9	7.5±4.0
Female	134 (56%)	78 (61%)	66 (52%)
FCI Play activities subscale (0–6)‡	3.3±1.7	3.8±1.4	3.4±1.6
FCI Play materials subscale (0–6)‡	2.1±1.1‡‡	2.2±1.0	2.2±1.2
Home observation subscale (0–11)‡	7.9±1.5	8.1±1.4	8.2±1.3
1+children’s book(s) present in home (n=288)‡	16 (11%)	9 (14%)§§	6 (8%)
MUAC <12.5 cm (n=296)‡	11 (8%)¶¶	6 (8%)§§	7 (9%)
Household characteristics			
Household size	5.2±2.2	5.3±2.6	5.2±2.0
Number of children 2–15 years	1.3 (1.2)	1.2 (1.1)*	1.2 (1.2)
Has cement floor	65 (22%)	28 (18%)	26 (16%)
Has brick walls	74 (25%)	27 (17%)	26 (16%)
Has electricity	243 (81%)	150 (94%)	139 (87%)
WASH			
Access to a handwashing station with water and soap or soapy water	62 (21%)	36 (23%)	33 (21%)
Access to a hygienic latrine§	102 (34%)	59 (37%)	43 (27%)
Use of potty‡¶ (n=297)	35 (24%)¶¶	16 (22%)	22 (28%)
Nutrition			
Maternal number of food groups	5.0±1.3	4.9±1.3	5.0±1.4
Maternal minimum dietary diversity**	182 (60%)	88 (55%)	101 (63%)
Child number of food groups (n=272)‡	3.8±1.5	3.7±1.4‡‡	4.1±1.4
Child minimum dietary diversity‡†† (n=272)	45 (35%)	19 (29%)‡‡	28 (37%)***

*n=159, 1 participant did not respond.

†Including index children born as of the baseline assessment.

‡Index children >6 months of age at baseline included (n=296, control=144, group=73, mixed=79), unless otherwise indicated.

§Clean, functional, Hygienic latrine. Government of Bangladesh National Sanitation Strategy, 2005 definition of hygienic latrine: Flush or pour-flush toilet/latrine to (1) Piped sewer system or (2) Septic tank; Pit latrine with slab and water seal; Pit latrine with slab and lid, no water seal; Pit latrine with slab and flap, no water seal; ventilated improved Pit latrine; composting latrine.

¶Use of potty for >50% of defecation events in last week.

**Mother reported eating 5 or more food groups in the last 24 hours, out of the following 10 groups: grains, roots and tubers, pulses, nuts and seeds, dairy products, animal flesh foods, eggs, dark green leafy vegetables, other vitamin A rich fruits and vegetables, other vegetables, other fruits.

††Children >6 months reported eating 5 or more food groups in the last 24 hours, out of the following groups: breast milk, grains, roots and tubers, legumes and nuts, dairy products, animal flesh foods, eggs, vitamin A rich fruits and vegetables, other fruits and vegetables.

‡‡n=143

§§n=66

¶¶n=145

***n=76

CES-D, Center for Epidemiologic Studies 20 Question Depression questionnaire, scores range from 0-60, with higher scores indicating more depressive symptoms experienced; FCI, Family Care Indicators, the play activities subscale is a sum score of the number of play activities that the caregiver participated in with the child in the previous three days (0-6), the play materials subscale is the number of varieties of play materials observed in the home, and reported that the child played with in the last 30 days (0-6); MUAC, Mid-upper arm circumference; WASH, water, sanitation and hygiene.

The mean number of the 18 sessions attended was similar across arms, with 14.2 (SD 4.0) in the group arm, and 15.4 (SD 3.2) in the combined arm. Participants in both intervention arms had a higher prevalence of any children’s picture books in the home at intervention endline (control 19%, group 85%, combined 93%), an indication that participants kept the books they received in intervention sessions.

### Home stimulation and child development outcomes

**Figure 2:**
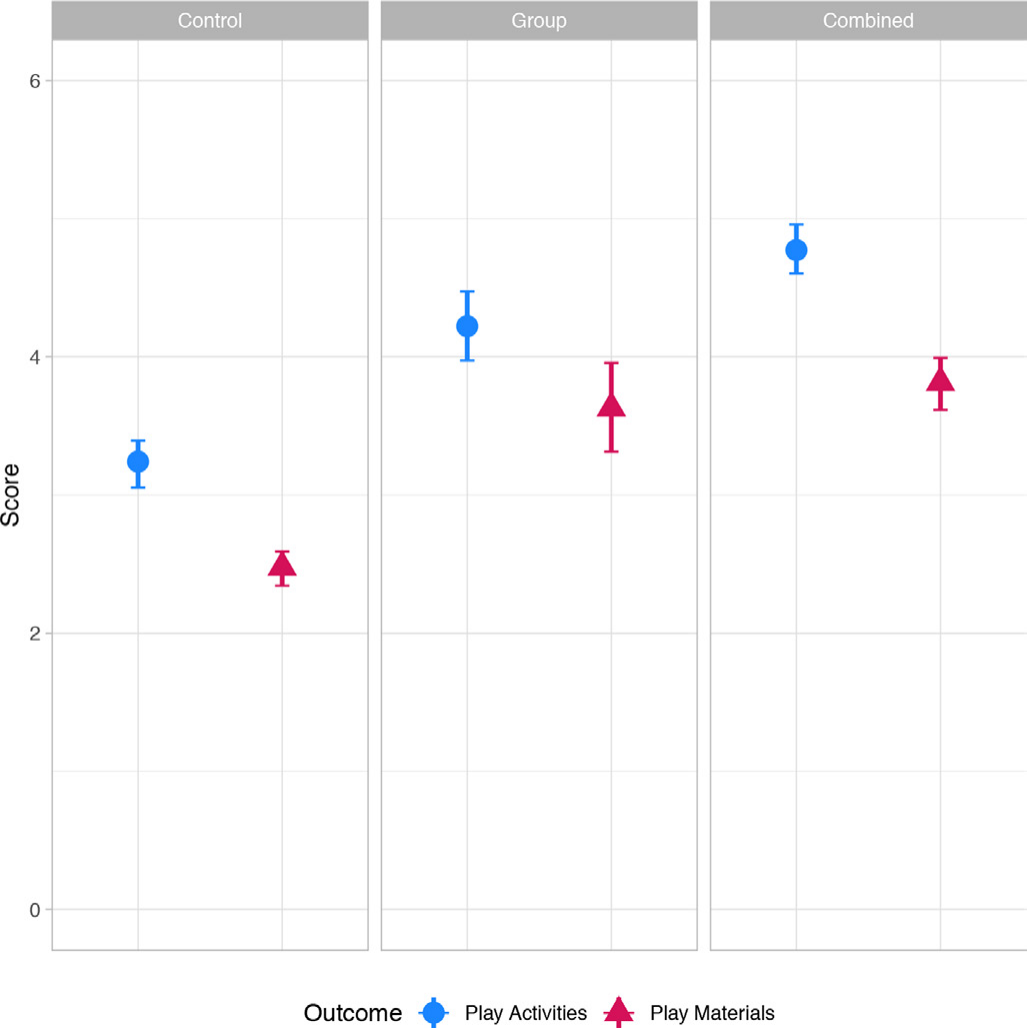
Mean stimulation in the home by study arm at endline. Points represent mean scores in each intervention arm, adjusted by child age at endline. Bars represent 95% CIs. Play activities (0–6): number of play activities that the primary caregiver engaged in with the child in the last 3 days. Individual items summed, and include: read books or looked at picture books; told stories; sang songs; took outside the home; played; named, counted or drew. Play materials (0–6): number of varieties of play materials observed in the home, and reported that the child played with in the last 30 days. Individual items summed, and include things: that play music; for drawing or writing; for pretending; used when running or jumping; for teaching shapes; for stacking.

Children in the group and combined intervention arms received significantly more stimulating activities in the past 3 days from their primary caregiver (age-adjusted means: group 4.22 (95% CI 3.97 to 4.47); combined 4.77 (4.60 to 4.96); control 3.24 (3.05 to 3.39)), had a larger variety of stimulating play materials in the home (age-adjusted means: group 3.63 (3.31 to 3.96); combined 3.81 (3.62 to 3.99); control 2.48 (2.34 to 2.59)) and had improved scores for the observation of caregiver responsiveness and the child’s environment scale (age-adjusted means: group 8.82 (8.59 to 9.10); combined 8.93 (8.67 to 9.18); control 8.26 (8.05 to 8.45)) when compared with the control arm (figure 2, unadjusted means and adjusted mean differences in table 3). For comparison with other work, we calculated the unadjusted Cohen’s d effect size for the stimulating caregiving activities outcome for the group (0.66 (0.45 to 0.87)) and combined (1.08 (0.87 to 1.29)) arms (results not shown). The stimulation activities ‘played with’, ‘read books to’ and ‘sang songs to’ were the stimulation activities that had the highest prevalence differences when comparing the intervention groups to the control (online supplemental figure S1).

**Table 3:** Child development, maternal mental health, nutrition, water, sanitation and hygiene and lead outcomes at endline

Indicators	Unadjusted mean±SD or N (%) by arm			Adjusted mean difference or risk difference versus control arm (95% CI)	
	Control	Group	Combined	Group	Combined
FCI					
Activities	3.2±1.5	4.2±1.5	4.8±1.3	1.05 (0.72 to 1.34)	1.56 (1.33 to 1.78)
Materials	2.5±1.4	3.64±1.7	3.9±1.6	1.18 (0.88 to 1.51)	1.36 (1.18 to 1.51)
Observation	8.3±1.5	8.8±1.2	8.9±1.5	0.56 (0.26 to 0.89)	0.67 (0.35 to 0.99)
ASQi					
Communication	56.0±16.4	58.4±15.3	58.6±16.3	0.32 (0.10 to 0.57)	0.21 (−0.04 to 0.49)
Fine motor	50.2±11.3	51.8±10.1	51.6±11.6	0.36 (0.11 to 0.63)	0.23 (−0.04 to 0.49)
Gross motor	58.6±15.1	60.8±14.1	59.3±16.1	0.27 (0.13 to 0.44)	0.04 (−0.18 to 0.22)
Problem-solving	55.6±15.8	56.9±14.6	58.2±15.6	0.18 (−0.04 to 0.43)	0.19 (−0.14 to 0.49)
Personal social	55.3±16.3	57.7±15.5	58.0±16.4	0.34 (0.10 to 0.63)	0.30 (−0.04 to 0.64)
Total	275.7±71.2	284.6±65.2	285.0±72.6	0.39 (0.16 to 0.64)	0.25 (−0.07 to 0.54)
CDI					
Receptive	44.8±23.7	49.0±22.4	49.0±23.3	0.25 (−0.04 to 0.55)	0.19 (−0.15 to 0.52)
Expressive	16.8±17.2	18.9±17.9	19.2±18.3	0.29 (0.06 to 0.50)	0.17 (−0.17 to 0.53)
Depressive symptoms					
CES-D score	15.0±9.0	13.2±7.0	14.1±9.1	−2.06 (−3.23 to −0.66)	−1.34 (−3.12 to 0.41)
Minimum dietary diversity					
Child*	146 (54%)	85 (61%)	98 (68%)	0.07 (−0.03 to 0.17)	0.14 (0.04 to 0.22)
Maternal†	177 (63%)	89 (63%)	113 (76%)	0.03 (−0.07 to 0.12)	0.14 (0.05 to 0.19)
WASH					
Hygienic latrine‡	96 (34%)	49 (35%)	51 (35%)	−0.03 (−0.19 to 0.11)	0.02 (−0.12 to 0.15)
Handwashing station with soap and water	59 (21%)	44 (31%)	36 (24%)	0.12 (0.01 to 0.24)	0.04 (−0.08 to 0.19)
Use of potty§	55 (20%)	42 (30%)	44 (30%)	0.13 (−0.02 to 0.28)	0.10 (0.00 to 0.21)
Lead					
Knowledge of lead	68 (24%)	103 (73%)	110 (74%)	0.51 (0.40 to 0.60)	0.52 (0.39 to 0.63)

Activities: number of play activities that the mother participated in with the child in the last three days (out of six). Materials: number of varieties of play materials observed available in the home (out of six). Observation: 11 observation items about caregiver-child interactions during the interview, and observations of the home environment (S5 for details)

For ASQi and CDI results: unadjusted mean values are raw values before standardisation, all adjusted mean differences use scores which are internally age-standardised to the control arm, and point estimates represent SDs from the control arm mean.

Adjusted analyses include the following potential covariates: interviewer (for FCI, ASQi and CDI outcomes), maternal and paternal education, child age, child sex, household income above the median, household wall material, presence of electricity in the home, the presence of household assets (wardrobe, table, chair, watch/clock, television, bicycle, sewing machine) and the measure assessed at baseline (if assessed in the whole population). Covariates with p<0.20 from a likelihood ratio test for each outcome are included in adjusted analyses.

n for ASQi and CDI outcomes (excluding outliers ± 4 SD from the control arm mean): communication n=566; fine motor n=559, gross motor n=563; problem-solving n=563; personal social n=550; total n=532; receptive n=573; expressive n=498 (only children over 9 months of age included).

*Children>6 months (n=555) reported eating 5 or more food groups in the last 24 hours, out of the following groups: breast milk, grains, legumes, dairy products, flesh foods, eggs, vitamin A rich fruits and vegetables, other fruits and vegetables (n=553).

†Mother reported eating 5 or more food groups in the last 24 hours, out of the following 10 groups: grains, legumes, nuts and seeds, dairy products, flesh foods, eggs, vitamin A rich fruits and vegetables, other vitamin A rich fruits and vegetables, other vegetables, other fruits.

‡Hygienic latrine (according to Government of Bangladesh National Sanitation Strategy 2005): Flush or pour-flush toilet/latrine to (1) piped sewer system, (2) septic tank; pit latrine with slab and water seal; pit latrine with slab and lid, no water seal; pit latrine with slab and flap, no water seal; ventilated improved pit latrine; composting latrine.

§Use of potty for >50% of defecation events for the index child in the last 7 days.

ASQi, Ages and Stages Questionnaire Inventory; CDI, Communicative Development Inventories; CES-D, Center for Epidemiologic Studies 20 question Depression scale; WASH, Water, Sanitation and Hygiene.

**Figure 3:**
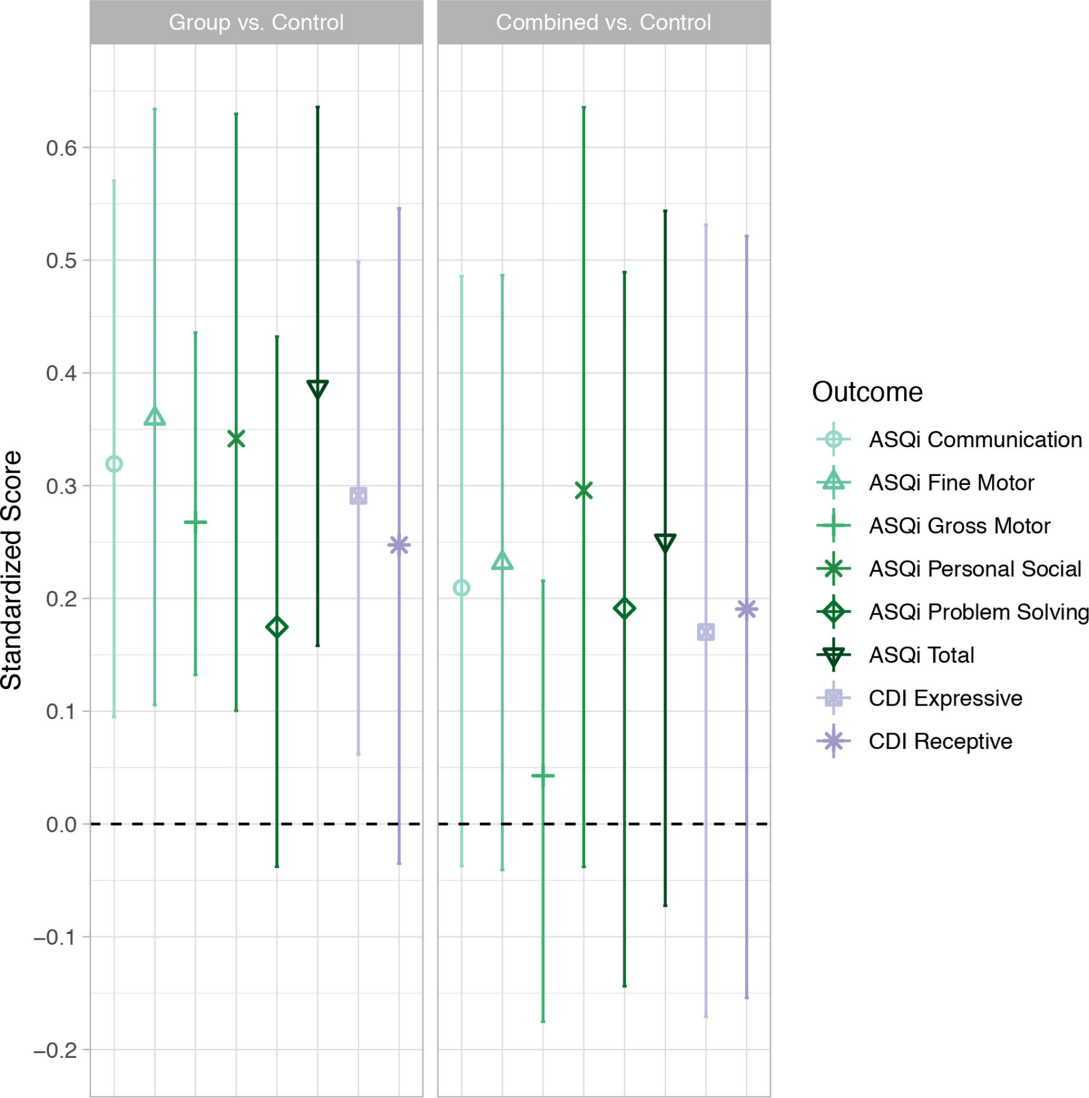
Adjusted mean differences in age-standardised Ages and Stages Questionnaire Inventory (ASQi) and Communicative Development Inventory (CDI), by intervention arm. Results for all domains are internally age-standardised to the control arm, points represent mean differences in standardised scores between each intervention arm and the control arm, lines represent 95% CIs. n by domain, after removing outliers and missing data: ASQi communication, n=566; ASQi fine motor, n=559; ASQi gross motor, n=563; ASQi problem-solving, n=563; ASQi personal social, n=550; ASQi total, n=532; CDI receptive n=573; CDI expressive n=498 (only including children over 9 months old).

Children in the group and combined arms scored higher than the control arm on all domains of the ASQi and CDI assessments. Differences for the group arm were significant for all domains except for problem-solving and receptive language, and differences for the combined arm were not significant for any domains (adjusted mean differences for standardised total ASQi score: group vs control 0.39 (0.16 to 0.64); combined vs control 0.25 (−0.07 to 0.54)) (figure 3, online supplemental table S5). In the supplementary analysis comparing both intervention arms to the control arm for the five domains of the Bayley-III assessment, the expressive communication and total Bayley-III scores were significantly higher for children in any intervention group compared to control (adjusted mean differences: expressive communication 0.33 (0.02 to 0.64); total Bayley-III score 0.38 (0.06 to 0.74; online supplemental figure S2).

### Maternal mental health, nutrition, WASH and lead outcomes

In both intervention arms, participants reported fewer depressive symptoms as compared with the control arm, indicated by lower CES-D scores, with a control arm mean of 15.01 (SD 8.96). The adjusted mean differences were significant for the group arm (−2.05 (95% CI −3.23 to 0.66)), but not the combined arm (−1.34 (-3.12 to 0.41); table 3). Minimum dietary diversity for mothers and children was improved in the combined intervention arm with adjusted prevalence differences of 0.14 (0.04 to 0.22) for mothers and 0.14 (0.05 to 0.19) for children. There were no significant differences between the group and control arms for maternal or child dietary diversity. There was no difference in the presence of a functional, clean and hygienic latrine for either intervention arm when compared with the control, and only the group arm had higher prevalence of a handwashing station with soap and water (adjusted prevalence difference: 0.12 (0.01 to 0.24)). Participants in both intervention arms had greater potty use when compared with the control, with differences significant for the combined arm (adjusted prevalence differences: combined 0.10 (0.00 to 0.21); group 0.13 (−0.02 to 0.28)). Knowledge of lead was significantly higher in both intervention arms, with a control arm prevalence of 0.24 and adjusted prevalence differences of 0.51 (0.41 to 0.61) and 0.52 (0.39 to 0.63) for the group and combined arm, respectively.

## Discussion

We found beneficial intervention impacts on our primary outcome of stimulation in the home, our secondary outcome of child development, as well as across a range of risk factors for child development addressed by the multicomponent intervention including caregiver depressive symptoms, caregiver and child dietary diversity, WASH and knowledge of lead. We observed impacts on play activities, play materials and observations of the home environment in both intervention arms. However, as the source of each play material was not asked, the results cannot be disaggregated by materials provided in the sessions and materials caregivers obtained on their own. Child development scores, as assessed by the ASQi and CDI were higher in both intervention arms when compared with the control, with differences for 6 of the 7 domains significant for the group arm, but not for the combined arm. Differences in standardised child development scores for the group intervention arm are between 0.18 to 0.39, similar to those from a group intervention in rural India which found significant improvements in cognition scores of 0.28 SDs,^28^ and an integrated home visiting programme in rural China, with intervention effects of 0.24 SDs.^13^ The results from a supplementary analysis on a subset of participants demonstrate improved receptive communication and total Bayley-III scores among those who received any intervention compared with the control. Though the current study was not powered to compare the group and combined arms directly, recent work from rural India finds similar effects on child development outcomes from group and individual home-based sessions.^28^ Further, recent work from rural Kenya indicates that group sessions may outperform combined delivery in some settings.^29^ Our work supports the delivery of multicomponent, mixed-age group sessions to improve risk factors for poor child development in rural Bangladesh.

Maternal depression is a risk factor for delayed child development, and has been associated with poor behavioural and developmental outcomes.^30^ We found fewer reported depressive symptoms from caregivers in both intervention arms, with a significant difference for the group arm. We hypothesise that the focus on maternal depression and the social support facilitated during the group intervention sessions contributed to the reduced depressive symptoms in both intervention arms. The effect may have been stronger in the group arm because this delivery mode offered structured peer social support 18 times over the course of the intervention compared with nine times in the combined arm. A meta-analysis of the effect of child stimulation interventions on caregiver depressive symptoms found no significant effect (−0.20 (−0.23 to 0.03)).^31^ However, the only group-based intervention that included contents on mental health found an effect size of −0.54 (−0.76 to −0.32)). This is higher in magnitude than the effect in the group arm of the current study (unadjusted Cohen’s d: −0.22 (−0.42 to −0.02)).^32^

We found improvements in nutrition, WASH and knowledge of lead in the intervention group. In the combined arm, a higher proportion of children and caregivers had a more diverse diet, though no difference was found for the group arm. As improving dietary diversity require changes in both purchasing and meal preparation, it may be that an approach where CHWs can respond to individual needs of families is required. There was no difference in presence of hygienic latrines in either intervention arm when compared with the control, more families in the group arm had a handwashing station with soap and water, and more caregivers in both arms reported that their child used a potty regularly, though the difference was only statistically significant for the combined arm. We do not know if the 17 families who were provided a potty would have purchased and used one in the absence of it being provided. We hypothesise that a subsidy may be required to improve hygienic latrine status in this low-income community given the investment required to upgrade WASH infrastructure. Caregiver’s knowledge of lead was improved in both arms, with large effect sizes. Lead is an invisible toxin unknown to the majority of the population at baseline, thus knowledge of lead is the first step towards reducing exposure. Secondary analyses will further investigate the intervention effects on behaviours related to lead exposure.

The unadjusted Cohen’s d effect sizes for stimulating caregiving practices in both intervention arms (group 0.66 (95% CI 0.45 to 0.87); combined 1.08 (0.87 to 1.29)) are slightly larger than the pooled effect sizes (0.57 (0.37 to 0.77)) from a recent meta-analysis of the effect of stimulation interventions on stimulation in the home, measured by the FCI and Infant Toddler Home Observation for Measurement of the Environment.^31^ The interventions in the meta-analysis included at most two additional components in additional to child stimulation, whereas our study included four additional components. Further, none of the interventions in the meta-analysis were delivered to both pregnant women and caregivers of children under 24 months of age. Thus, our findings suggest that the effects on caregiver-related outcomes were not diminished with the inclusion of multiple integrated intervention components, nor intervention delivery across both pregnant women and caregivers of young children.

In the RINEW intervention, groups were based on geographical proximity to reduce barriers to attendance, and included pregnant women and mixed-age, caregiver-child dyads. Other group-based ECD interventions are delivered to groups with children of similar ages, to allow for the presentation of age-specific materials relavent to the whole group.^13^ In addition to reducing barriers to attendance, grouping participants with others who they may interact with daily may increase the potential for continued social support for intervention activities outside sessions. However, delivering sessions to a mixed-age group may increase session duration, and include less engaging components for some participants (ie, pregnancy contents for non-pregnant participants).^33^ In future work, the tradoffs of mixed-age delivery and geographic proximity should take into account the geographic density of eligible participants and accessability of session locations.

This study has several strengths, including the focus on a group-based intervention to address multiple risk factors for poor ECD, which makes this approach more scalable than one-on-one home visiting programmes. In addition, the intervention was delivered simultaneously to both pregnant and lactating women with children of mixed-ages, an approach that is easier to scale than more narrowly focused programmes. We used a tablet application to facilitate session delivery, which enabled the inclusion of multiple age-specific intervention components. Another unique feature of this programme was the integration of information on the reduction of heavy metal exposure, in addition to standard messages about nutrition, health and hygiene. Finally, we included a set of outcomes that spans a broad range of influences in early life and development in order to gain a more comprenensive understanding of the impact of the intervention on a child’s development and well-being.

The current study has important limitations. First, due to budget constraints, the sample was not powered to detect small differences on many of the secondary outcomes, or differences between the two active intervention arms. As such, we are only able to interpret the direction and magnitude of these effects. Second, the FCI, ASQi, CDI, dietary diversity, depressive symptoms and knowledge of lead assessments are primarily based on caregiver-report, allowing for the possibility that caregiver responses about behaviours could be influenced by knowledge and social desirability, or caregivers’ mental health status. The risk of respondent bias was minimised through extensive training of survey enumerators, the use of direct-observation items within the ASQi, an observation scale to complement the FCI and follow-up questions to confirm reported lead knowledge. We found significant improvements in observed caregiver responsiveness and the caregiving environment, highlighting that changes were found for observed behaviour in addition to caregiver report. Additionally, as this was the first time implementing such an intervention curriculum in Bangladesh, there were some adjustments to the strategies used to build group cohesiveness and encourage attendance, and intervention modules were refined as the sessions progressed. The results do not represent the impacts of the intervention that may be achieved with further refinements, and the current estimates may be a lower bound on the possible impact. Finally, we were not able to examine the cost effectiveness of group compared with individual or combined delivery mechanisms for integrated interventions.

This intervention illustrates the feasibility of locally recruited CHWs delivering a group-based, mixed-age, multicomponent ECD intervention in rural Bangladesh. The feasibility of scaling such a group-based intervention through a government health system, or the large-scale implementation through a regional or national non-government organisation is not known, and warrants exploration. A promising, recent study found that child stimulation sessions delivered through Government of Bangladesh community clinics to pairs of mother-child dyads, had large impacts on child development.^34^ Notably, these clinics serve as regular point of care, routinely providing maternal and neonatal healthare as well as nutrition and health education. Differences in CHW workload, session attendance and intervention impacts with each of the delivery mechanisms will inform the design of scalable and impactful child development interventions.

The long-term impact of the RINEW intervention, or similar integrated interventions targeting multiple risk factors for ECD, will be critical to understand the scope of intervention impact. Although many stimulation interventions have shown impacts on child development outcomes at intervention endline, there is mixed evidence on the later impacts of these early interventions.^17 35^ It is possible that integrated interventions addressing multiple risk factors may contribute to sustained intervention impacts on child development as they more holistically improve children’s early-life caregiving and health environments. Medium and long-term follow-up of children enrolled in multicomponent interventions is required to examine this hypothesis.

## Conclusion

In conclusion, we found that a carefully designed group-based multicomponent intervention delivered by well-trained CHWs can address multiple additional risk factors for child development beyond stimulating caregiving, and demonstrate similar effects on stimulating caregiving as interventions with fewer integrated components. CHWs were able to deliver the complex multicomponent RINEW intervention for 9 months and community members regularly attended intervention sessions regardless of delivery platform. This multicomponent approach may be used as a template to design a scalable and impactful intervention to improve child well-being in low-income settings.
