# Supplementary material for: A holistic approach to promoting early child development: a cluster randomised trial of a group-based, multicomponent intervention in rural Bangladesh
Source: BMJ Glob Health. 2021 Mar 16;6(3):e004307. doi: 10.1136/bmjgh-2020-004307 (PMC7970287; doi:10.1136/bmjgh-2020-004307)
Supplement: Supplementary data [file bmjgh-2020-004307supp001.pdf]

Supplementary material for A holistic approach to promoting early child development: a cluster randomised trial of a group-based, multicomponent intervention

Table S1: Exceptions to inclusion criteria for village selection

# of villages	Exception
**1**	Household ownership of 92.2% which was less than the mean -1.5*SD = 93.4%.
**1**	Village that had 193 households at the 2011 BBS census.
**2**	Village centers 1.15 km apart

For villages that were less than 1km apart we kept the village that was closer to the mean values on basic demographic variables. When three village centers were within 1 km of each other, we exluded the village in the middle, so that the remaining villages were approximately 2 km apart.

Table S2: Brief description of intervention components by session (Group arm)

Session	Contents
G1	• Recap of community meeting (how WASH, nutrition, and child stimulation can help child development)<br>• Importance of early stimulation<br>• Introduction to thinking healthy<br>• Interactions with baby
G2	• ANC<br>• Importance of MMN/MNP/LNS<br>• Maternal and Child nutrition, growth, and development<br>• Interactions with baby<br>• Talking while bathing baby<br>• Distribution of micronutrients according to age/status
G3	• Sanitation, infection, growth and development<br>• Water quality, infection, growth and development<br>• Early care of neonate and cord care<br>• Hand washing, infection, growth and development<br>• Food hygiene, infection, growth and development<br>• Interactions with baby<br>• Talking while feeding the baby<br>• Distribution of kangaroo care foot measuring stick to pregnant women
G4	• Pleasurable activities and reduced maternal workload<br>• Physiological symptoms of pregnancy<br>• Interactions with baby<br>• Learning common nouns<br>• Thinking healthy regarding “Mother’s health”<br>• Distribution of micronutrients according to age/status
G5	• Maternal dietary diversity<br>• Maternal weight gain<br>• Interactions with baby<br>• Talking while walking with baby<br>• Lead information

Pitchik HO, *et al. BMJ Global Health* 2021; 6:e004307. doi: 10.1136/bmjgh-2020-004307

G6	• Breastfeeding<br>• Kangaroo Mother Care<br>• Love and praise baby<br>• Interact while breast feeding<br>• Interactions with baby<br>• Learning action words<br>• Thinking healthy regarding "Mother's relationship with baby"<br>• Distribution of micronutrients according to age/status
G7	• Kangaroo mother care<br>• Exclusive Breastfeeding<br>• Breastfeeding challenges<br>• Interactions with baby<br>• Learning body parts
G8	• Safe disposal of human and animal feces<br>• Constructing a hygienic latrine<br>• Interactions with baby<br>• Talking while dressing baby<br>• Thinking healthy regarding "Mother's relation with people around her"<br>• Distribution of micronutrients according to age/status
G9	• Use of child potty and potty training<br>• Handwashing<br>• Interactions with baby<br>• Learning to follow directions
G10	• Child dietary diversity<br>• Processed food<br>• Responsive feeding<br>• Interactions with baby<br>• Touch and bring game<br>• Thinking healthy regarding "Mother's health"<br>• Distribution of micronutrients according to age/status
G11	• Handwashing, especially before feeding<br>• Making soapy water<br>• Interactions with baby<br>• Talking while doing household activity<br>• Sick child feeding
G12	• Child feeding frequency<br>• Continued breast feeding<br>• Responsive feeding<br>• Interactions with baby<br>• Funny game-act out activities<br>• Thinking healthy regarding "Mother's relation with baby"<br>• Distribution of micronutrients according to age/status
G13	• Water storage<br>• Food storage<br>• Exclusive breastfeeding<br>• Continued breastfeeding<br>• Interaction with baby

	• Finger game
G14	• Handwashing<br>• Soapy water<br>• Interaction with baby<br>• Peek-a-boo game<br>• Thinking healthy regarding “Mother’s relationship with people around her”<br>• Adverse health effect of arsenic on human health<br>• Collect and drink water from arsenic free safe water source<br>• Distribution of micronutrients according to age/status
G15	• Interaction with baby<br>• Hand game<br>• Sick child Feeding<br>• Complementary feeding<br>• Construct/upgrade and maintain child friendly toilet<br>• Teach and encourage children elder than three years to use child friendly toilet
G16	• Integrated recommendation on food storage, hand washing, water storage<br>• Exclusive breastfeeding,<br>• Continued breastfeeding<br>• Maternal and Child Dietary Diversity<br>• 1000 days<br>• Interaction with baby<br>• Mirror game<br>• Thinking healthy-real life experiences<br>• Building collective efficacy avoid arsenic contamination<br>• Distribution of micronutrients according to age/status
G17	• Healthy interaction process, centered around feeding<br>○ Complementary feeding, processed food, sick child feeding,<br>○ Handwashing and responsive feeding<br>○ Healthy Thinking<br>• Importance of early stimulation<br>• Interaction with baby<br>• Find-it-game
G18	• Healthy Community<br>○ Healthy Thinking<br>○ Whole community practices healthy behaviors<br>• Recap of community meeting (Mention how WASH, nutrition, and child stimulation can help child development)<br>• Interactions with baby<br>• Learning action words

Table S3: Observation items during the FCI interview

	Observation item
1.	(Mother/Guardian) Spontaneously spoke to child twice or more (excluding scolding)
2.	(Mother/Guardian) Responded verbally to child's speech or verbal bids for attention
3.	(Mother/Guardian) Provided toys or interesting activities for child
4.	(Mother/Guardian) Caressed, kissed, or hugged child at least once
5.	(Mother/Guardian) Kept child in view/could see child/looked at (him/her) often
6.	*(Mother/Guardian) Interfered with child's actions or restricted child from exploring more than 3 times
7.	Child's play environment is safe (no potentially dangerous health or structural hazards within a toddler's or infant's range)
8.	Reading material (newspapers, magazines, etc.) is present and visible
9.	Child and child's clothing appear clean
10.	*(Mother/Guardian) Slapped or spanked child one or more times
11.	*There is evidence that older children are handling the child inappropriately (i.e., handling roughly, hitting, etc.)

All questions have a binary yes/no response, and the total score is the sum of all items, with negative items reverse coded; Items responses are summed with starred items are reverse coded. Items are a subset of observation items from the Early Childhood HOME assessment that have been piloted, pretested, and previously used in Bangladesh, in addition to two additional items (questions 9 and 11).

Table S4: Number of direct assessment items by ASQi domain

Domain	Direct assessment items	Total items
Communication	6	64
Gross Motor	11	65
Fine Motor	20	63
Problem Solving	9	68
Personal Social	4	50

Pitchik HO, *et al. BMJ Global Health* 2021; 6:e004307. doi: 10.1136/bmjgh-2020-004307

Table S5: Internally age-standardized child development outcomes at endline

	mean (SD)	
	**Group**	**Combined**
**ASQi**		
Communication	0.29 (0.87)	0.19 (0.96)
Fine Motor	0.26 (0.91)	0.15 (1.07)
Gross motor	0.24 (0.88)	-0.01 (1.11)
Problem-solving	0.13 (0.96)	0.19 (0.97)
Personal Social	0.27 (0.99)	0.24 (1.18)
Total	0.29 (0.92)	0.19 (1.09)
**CDI**		
Receptive	0.26 (0.93)	0.21 (0.91)
Expressive	0.25 (1.15)	0.16 (1.22)

Scores are internally age-standardized to the control arm: control arm means are between -0.02 and 0.02, and standard deviations are between 0.99-1.00, and not included in the table.
n by outcome (excluding outliers ± 4 SD from the control arm mean): Communication n=566; Fine Motor n=559, Gross Motor n=563; Problem-solving n=563; Personal Social N=n=550; Total n=532; Receptive n=573; Expressive (only children over 9 months of age included) n=498

Figure S1: Play activities in the home, by activity, at endline

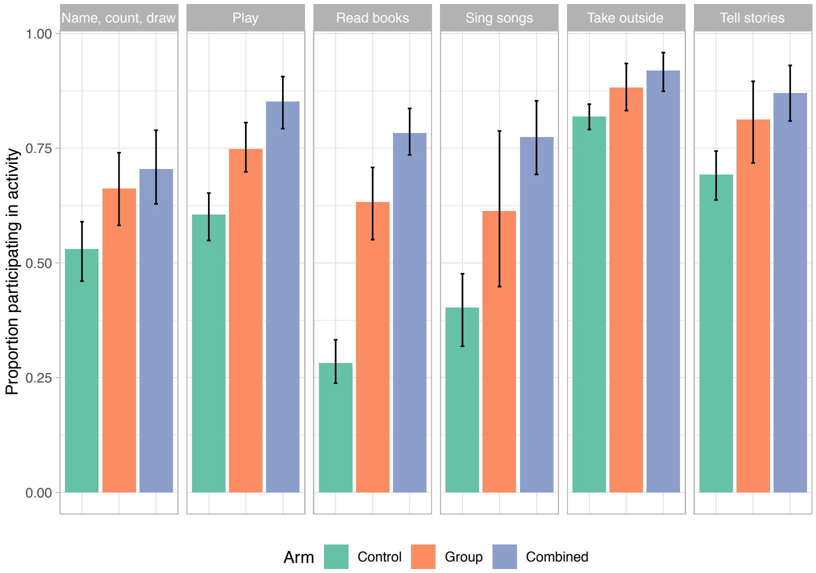

Each of the six stimulation activities is caregiver reported participation in the previous three days

Figure S2: Adjusted mean differences in age-standardized Bayley-III scores, any intervention vs. control

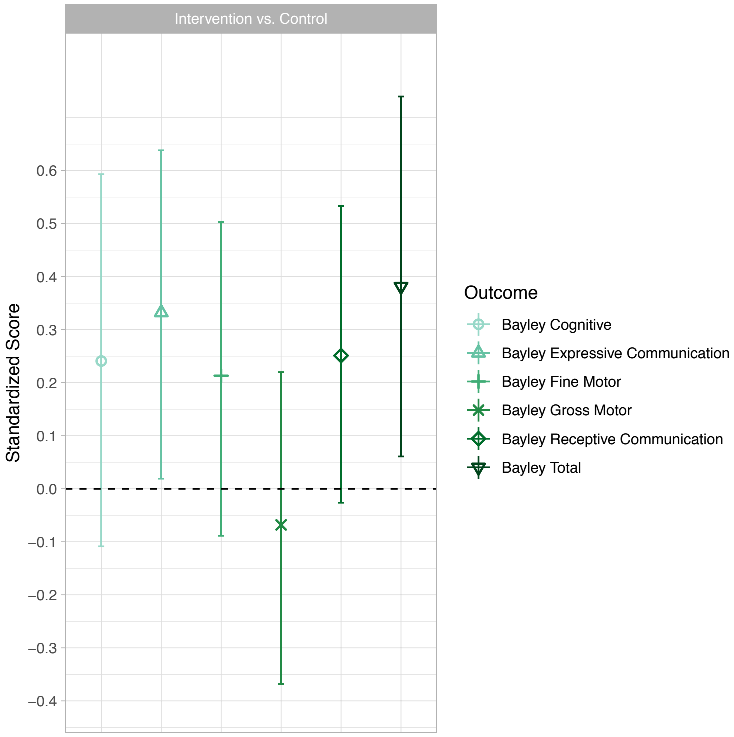

All results are internally age-standardized to the control arm, and point estimates represent differences between any intervention group, and the control arm. Lines represent 95% confidence intervals
n by domain, after removing outliers (± 4 SD) and individuals with missing data on each domain: Expressive Communication, n=250; Receptive Communication, n=252; Gross Motor, n=250; Fine Motor, n=249; Cognitive, n=249; Total, n=245
Potential covariates for inclusion in adjusted include interviewer, maternal and paternal education, child age, child sex, household income above the median, household wall material, presence of electricity in the home and the presence of household assets (wardrobe, table, chair, watch/clock, television, fridge, bicycle, sewing machine). For each outcome covariates were prescreened using a likelihood ratio test, and all covariates with $p<0.20$ were included in adjusted analyses.
